# Evidence-Informed Deliberative Processes for Universal Health Coverage: Broadening the Scope: Comment on "Priority Setting for Universal Health Coverage: We Need Evidence-Informed Deliberative Processes, Not Just More Evidence on Cost-Effectiveness"

**DOI:** 10.15171/ijhpm.2016.148

**Published:** 2016-11-27

**Authors:** Unni Gopinathan, Trygve Ottersen

**Affiliations:** ^1^Oslo Group on Global Health Policy, Department of Community Medicine and Global Health and Centre for Global Health, University of Oslo, Oslo, Norway.; ^2^Department of International Public Health, Norwegian Institute of Public Health, Oslo, Norway.

**Keywords:** Universal Health Coverage (UHC), Priority Setting, Evidence-Informed Deliberative Processes, Public Health Interventions

## Abstract

Universal health coverage (UHC) is high on the global health agenda, and priority setting is fundamental to the fair and efficient pursuit of this goal. In a recent editorial, Rob Baltussen and colleagues point to the need to go beyond evidence on cost-effectiveness and call for evidence-informed deliberative processes when setting priorities for UHC. Such processes are crucial at every step on the path to UHC, and hopefully we will see intensified efforts to develop and implement processes of this kind in the coming years. However, if this does happen, it will be essential to ensure a sufficiently broad scope in at least two respects. First, the design of evidence-informed priority-setting processes needs to go beyond a simple view on the relationship between evidence and policy and adapt to a diverse set of factors shaping this relationship. Second, these processes should go beyond a focus on clinical services to accommodate also public health interventions. Together, this can help strengthen priority-setting processes and bolster progress towards UHC and the Sustainable Development Goals.

## Background

Universal health coverage (UHC) is high on the global health agenda, and priority setting is fundamental to the fair and efficient pursuit of this goal. In a recent editorial, Rob Baltussen and colleagues point to the need to go beyond evidence on cost-effectiveness and call for evidence-informed deliberative processes when setting priorities for UHC.^1^ The starting point is that multiple priority-setting criteria are important alongside cost-effectiveness, including priority to the worse off and financial protection.^2,3^ Since stakeholders are likely to disagree about the exact content and the relative importance of substantive criteria, deliberation among all relevant stakeholders are pivotal to ensure that they and the values and beliefs these bring to the table are considered in the priority-setting process.^4^ At the same time, these processes must be based on rational decision-making in the form of evidence-informed evaluation. Correspondingly, Baltussen and colleagues see “evidence-informed” and “deliberation” as the two essential elements in achieving legitimacy in priority setting.^1^

We agree that processes of this kind are crucial at every step on the path to UHC, and hopefully the years ahead will bring intensified efforts to develop and implement such processes. However, if this does happen, it will be essential to ensure a sufficiently broad scope in at least two respects. First, the design of evidence-informed priority-setting processes needs to go beyond a simple view on the relationship between evidence and policy and adapt to a diverse set of factors shaping this relationship. Second, the processes should go beyond a focus on clinical services to accommodate also public health interventions.

## Evidence to Policy

Baltussen and colleagues affirm that priority setting is a political process and mention bargaining among different lobbies. This hints at the complex relationship between evidence and policy; a relationship that is forged by a diverse set of factors. To be effective, priority-setting processes need to be adequately tailored to these.

The probably simplest view on evidence-informed policy is that researchers just need to publish their findings and then policy-makers will pick them up and craft their policies accordingly. While some of today’s practice and behavior seem to align with this view, few, if anyone, profess such a view. Moreover, a sizable body of knowledge now exist about strategies that go beyond the “simplest view” and can help translate evidence into policy.^5,6^ On the evidence-supply side, an evidence-informed process can be promoted by conducting research that speaks directly to the needs of the policy-making process, communicating this research effectively, making the research evidence available in a timely manner, and convening deliberative dialogues that are informed by research evidence.^5^ On the evidence-demand side, strategies include creating institutional mechanisms that privilege the use of research evidence, demanding effective communication of high-quality, locally relevant and actionable research evidence, and creating prompts for the use of research evidence in decision-making.^5^ One example of the latter is the mandatory completion of an evidence checklist, documenting where evidence was searched for, what types of evidence were found, and how evidence was used before a decision was made.^5^ These strategies can all contribute to improving the interaction between researchers and policy-makers, and this factor was identified by a recent systematic review to be one of the most important ones for increasing the likelihood of policy-making being evidence-informed.^6^

While these strategies clearly go beyond the “simplest view,” many critical commentators still deem them too narrow and too rooted in a linear model, where policies are seen as the direct result of a “chain of research, evidence, and recommendations.”^7^ It is claimed that these strategies fail to account for the truly complex relationship between evidence and policy and for the diverse set of factors shaping it; factors that are better captured by other explanatory models.^7-10^

This landscape of models of policy change has been reviewed by Katherine Smith as part of a large body of work examining the relationship between public health research and policy.^9^ She highlights four additional factors—framed as ways of conceptualizing the relationship between evidence and policy beyond a linear model—that affects how evidence informs policy. First, evidence can inform policy through policy entrepreneurs (who may be researchers or an actor engaged by researchers), who exploit policy windows to craft and put forward evidence-informed policy solutions. Second, evidence can inform policy when it appeals to the normative interests and ideological positions of the stakeholders involved (conversely, evidence risks being disregarded if it challenge prevailing norms and ideology). Third, evidence can inform policy when it contributes to shifting how an issue is conceptualized and perceived. Finally, evidence can inform policy through research-informed ideas, including broader concepts describing how a policy problem should be addressed.

While much of this literature on how evidence informs policy is geared towards broad policies, it comes with important insights for priority setting, and somewhat similar analyses have been done also for priority-setting processes more directly.^11-13^ Frameworks like the frequently applied Shiffman and Smith framework for analyzing determinants of political priority for global health issues can also help better understand how priority-setting processes best can be designed.^14^

A more comprehensive view on how evidence informs policy can also help demonstrate why priority-setting processes for UHC need to be firmly positioned within robust institutions.^15^ Priority setting in health is characterized by a rapidly evolving evidence base, controversy over values, a demand for consistency to ensure fairness, and the involvement of a multitude of stakeholders, many of which have strong political or financial interests in how priorities are set. These circumstances suggest that any poorly institutionalized priority-setting process will struggle to ensure an effective and fair path towards UHC, and especially a path that is sensitive to the voices of the poor and marginalized. It is, therefore, timely that new initiatives, such as the International Decision Support Initiative (iDSI), are working to strengthen institutions for priority setting at national and global levels.^16^

## Public Health Interventions

Baltussen and colleagues do not specify exactly what kinds of interventions are to be considered through the envisaged processes. However, they have earlier described how multi-criteria decision analysis has been used to evaluate broad interventions such as smoking cessation programs and alcohol taxation.^17,18^ Multi-criteria decision analysis considered by Baltussen and colleagues to be integral to an evidence-informed deliberative process.^1^ This suggests that the processes Baltussen and colleagues propose can address public health interventions, understood as population-based, preventive measures. In addition to taxes and subsidies (eg, levy on alcohol), such interventions include laws and regulations (eg, ban on promotion of tobacco), informational campaigns (eg, ads about the benefits of healthy diet), and improvements in the built environment (eg, construction of safe roads). Most of the discussion on priority setting in health have, however, focused on services targeted at individuals and often clinical services. Similarly, discussions on UHC tend to concentrate on such services.^19,20^ Thus, there is a “double danger” that the future development of priority-setting processes for UHC will pay limited attention to public health interventions.

Given the importance of public health interventions for population health outcomes and the wider Sustainable Development Goals, priority-setting processes should plausibly be designed in a way that can accommodate also such interventions. These interventions, however, differ from individual, clinical services in multiple ways that can influence how these interventions should be assessed.

First, many public health interventions provide economic or educational benefits alongside health benefits. School feeding programs, for example, tend to improve energy intake and micronutrient status but also school attendance.^21^ This does not call for one comprehensive metric subsuming all kinds of relevant outcomes, as this is currently impracticable. However, it does call for an evaluative process in which non-health benefits are systematically screened for, systematically assessed if found to be potentially substantial, and systematically exhibited to the relevant decision-makers.

Second, many public health interventions require collaboration and often co-financing across sectors. Efforts to reduce air pollution, for example, normally requires involvement of the environmental and transport sector. The evaluation process itself can facilitate inter-sectoral collaboration and financing by exposing benefits accruing outside the health sector, by including stakeholders from other sectors in the process, and by linking evaluation to various forms of joint budgeting.^22^ Third, public health interventions can often have substantial impact on health inequalities, through the social determinants of health or more directly. Increases in tobacco price, for example, tend to have a pro-equity effect on socioeconomic disparities in smoking.^23^

Overall, this suggests that for priority-setting processes to adequately accommodate public health interventions, they need to take non-health outcomes into account, include non-health stakeholders in the deliberations, and capture distributional impacts, all of which are done only to a limited extent today. At the same time, the special features of public health interventions may generate dynamics for evidence-informed policy-making quite different from those of clinical services. This difference is partly generated, partly reinforced by the fact that public health interventions, compared to clinical services, have a more sparse and qualitatively different evidence base, more often require large structural changes and changes pertaining to opposing ideals and ideologies, and typically engage different interests groups, with less involvement by the pharmaceutical industry or patient advocacy groups. This, in turn, just underscores the importance of tailoring priority-setting processes to the diverse set of factors shaping the relationship between evidence and policy.

## Way Forward

Development and implementation of evidence-informed deliberative processes for UHC will hopefully take off over the coming years. With a sufficiently broad scope, these efforts can accelerate progress towards UHC and the Sustainable Development Goals.

## Ethical issues

Not applicable.

## Competing interests

Authors declare that they have no competing interests.

## Authors’ contributions

Both authors contributed equally to the writing of this paper.

## Authors’ affiliations

^1^Oslo Group on Global Health Policy, Department of Community Medicine and Global Health and Centre for Global Health, University of Oslo, Oslo, Norway. ^2^Department of International Public Health, Norwegian Institute of Public Health, Oslo, Norway.
